# Posttraumatic Growth in Postlingually Deaf Patients With Cochlear Implants: The Effect of Stress-Coping Strategies, Sociodemographics, and Deafness-Related Factors

**DOI:** 10.3389/fpsyg.2021.546896

**Published:** 2021-08-17

**Authors:** Joanna Kobosko, W. Wiktor Jedrzejczak, D. Beata Porembska, Anna Geremek-Samsonowicz, Henryk Skarzynski

**Affiliations:** ^1^Institute of Physiology and Pathology of Hearing, Warsaw, Poland; ^2^World Hearing Center, Nadarzyn, Poland; ^3^The Maria Grzegorzewska University, Warsaw, Poland

**Keywords:** posttraumatic growth, postlingual deafness, stress coping strategies, gender differences, cochlear implant

## Abstract

The aim of this study was to assess whether cochlear implant (CI) users who had been postlingually deaf developed a more positive outlook on life—the so-called posttraumatic growth (PTG)—as a result of their disability and to examine how PTG related to their stress-coping strategies and personal circumstances. The study group consisted of 119 postlingually deaf CI users. The participants were asked to anonymously fill in several questionnaires: the Post-Traumatic Growth Inventory (PTGI), Coping Orientations to Problems Experienced (Brief COPE), and a form asking for personal details and factors related to their deafness and CI use. The PTG of postlingually deaf CI users was similar to that found in people with other severe health problems. The time that had elapsed since the hearing was lost and the time from receiving a CI were positively correlated with PTG. The level of PTG was correlated with the particular coping strategies used and differed between men and women. We found that the development of PTG could emerge from both approach-oriented coping strategies (e.g., active coping and planning) and avoidance-oriented coping strategies (e.g., denial, self-distraction, and self-blame). Paradoxically, the avoidance strategies could play a positive role in the development of PTG. This reinforces the idea, previously raised in the PTG literature, that such strategies exert a defensive and protective function—an “illusory” side of PTG—which operates together with the positive constructive side, and both help develop the sense of well-being of a person.

## Introduction

Postlingually acquired deafness or profound hearing loss is a highly stressful life event, which usually arrives as a severe shock in the life of a person (Hallberg and Carlsson, 1993; Kerr and Cowie, 1997; Heydebrand et al., 2005; Hallam et al., 2006; Hallberg et al., 2008; Kashubeck-West and Meyer, 2008; Du Feu, 2014; Kobosko, 2015; Williams et al., 2015). Whether someone loses hearing gradually or suddenly—-for example, as a result of illness or accident—-it is likely to be an adverse/traumatic experience (Du Feu and Fergusson, 2003). However, when we look at it from the perspective of positive psychology, people who lose their hearing have the chance to experience positive changes as a result of this impact on their life—-a change that is referred to as posttraumatic growth (PTG). This theoretical concept suggested by Tedeschi and Calhoun (2004), Calhoun and Tedeschi (2014), and Martz and Livneh (2016) has been backed up by numerous studies on people with chronic illness or disabilities (CID) and by systematic reviews (Barskova and Oesterreich, 2009; Harding et al., 2014; Koutrouli et al., 2014; Ogińska-Bulik, 2015; Rzeszutek and Gruszczyńska, 2018; Wu et al., 2019). However, the phenomenon of PTG has not yet been studied in a population of postlingually deaf people, i.e., those who have experienced hearing loss following their acquisition of speech and language.

The difficulties experienced by someone who has postlingually lost hearing relate not only to day-to-day speech and limitations in verbal communication but also to longer-term difficulties in interpersonal relations with family, friends, and work colleagues. The problems can extend to various social situations, which can be highly negative, sometimes amounting to stigmatization (Kerr and Cowie, 1997; Kashubeck-West and Meyer, 2008; Du Feu, 2014; Williams et al., 2015). Such difficulties often produce high levels of psychological distress, depression, and anxiety (Hallam et al., 2006; Fellinger et al., 2012; Du Feu, 2014), and lead to lowered satisfaction with life and general loss of well-being (Kerr and Cowie, 1997; Heydebrand et al., 2005; Kashubeck-West and Meyer, 2008; Kobosko, 2015; Williams et al., 2015).

People who become postlingually deaf can benefit from a cochlear implant (CI) (Hallberg and Ringdahl, 2004; Heydebrand et al., 2005; Lassaleta et al., 2006; Kennedy et al., 2008; Rembar et al., 2009; Rostkowska et al., 2012; Ramos-Macías et al., 2016). This is a device that is surgically implanted into the head and transforms sound into electric stimuli that are in turn transmitted to the nervous system (Lazard et al., 2012; Blamey et al., 2013). It provides a new way of perceiving sounds, and most importantly speech. In Poland, CIs have been available since 1992 (Skarżyński et al., 1993), and many studies have shown that they improve health-related quality of life.

As we know from our clinical practice, when deafness first occurs it is perceived by the patient as “something terrible,” “a collapse of the world,” “a black abyss,” or a “situation without exit.” However, in light of the positive psychology just mentioned, they may also experience PTG—-positive changes in various areas of their life (although not all patients do so). When a patient with postlingual profound hearing loss is first provided with a CI, the device and the ability to hear again help them adapt to their deafness. Hence, cochlear implantation is perceived by most CI users as a positive (not an adverse) experience—as can be indirectly concluded from the reports of postlingually deaf CI users and their significant others (Hallberg and Ringdahl, 2004; Heydebrand et al., 2005; Kennedy et al., 2008). Our own years of experience of working with such patients confirms that view. However, the complication is that a CI cannot, by itself, be treated as a source of PTG. In fact, a CI may indirectly promote PTG as the by-product of a particular stress-coping strategy.

To date, only a few researchers have looked at the positive aspects of hearing loss. For example, Kerr and Cowie (1997) and Stephens and Kerr (2003) found that the experience of acquired deafness can enrich life by contributing to, among other things, “developing a better understanding of human nature.” Some people with hearing loss have done so with great success (Manchaiah et al., 2015), but it is not known whether, when finding positive aspects to their hearing loss, they also experienced PTG, because not every positive change indicates the development of PTG.

To the best of our knowledge, there has been no research on the PTG that may occur in the personal development of postlingually deaf people who have also become CI users.

### Posttraumatic Growth and Chronic Illness or Disability

In order to understand how trauma might lead to possible positive outcomes, Tedeschi and Calhoun (2004) and Calhoun and Tedeschi (2014) introduced the concept of PTG. Their concept posits that an encounter with trauma can become the source of a highly positive change in a person. The way the PTG concept is used here is that the trauma of a subject is synonymous with a “crisis (or) major stressor (which) describe circumstances that significantly challenge or invalidate important components of the individual's assumptive world” (Tedeschi and Calhoun, 2004; Calhoun and Tedeschi, 2014). It is a “seismic event.” The authors of the PTG concept emphasized that PTG is an inevitable concomitant of how trauma is coped with, not a secondary mechanism that an individual calls on in order to cope. Importantly, PTG involves the appearance of positive changes which go beyond the level of psychological functioning of a person which existed prior to the trauma (Calhoun and Tedeschi, 2014). Of course, PTG can also be accompanied by psychological distress (Tedeschi and Calhoun, 2004; Calhoun and Tedeschi, 2014).

Research on PTG in the domain of health has mainly been carried out on patients with chronic, serious, and sometimes incurable diseases such as cancer (Cordova et al., 2001; Steel et al., 2008; Barskova and Oesterreich, 2009; Jansen et al., 2011; Scrignaro et al., 2011; Harding et al., 2014; Koutrouli et al., 2014; Andysz et al., 2015; Mazurkiewicz et al., 2015; Ogińska-Bulik, 2015), heart problems (Barskova and Oesterreich, 2009; Łosiak and Nikiel, 2014; Ogińska-Bulik, 2015; Juczyński and Ogrodnik, 2017), and AIDS/HIV (Barskova and Oesterreich, 2009; Calhoun and Tedeschi, 2014; Ogińska-Bulik, 2015; Rzeszutek et al., 2016; Rzeszutek and Gruszczyńska, 2018). Less often, PTG has been applied to people with permanent health losses such as those with spinal cord injuries (Ogińska-Bulik, 2015; Byra, 2016). Research has uncovered strong associations between PTG and health levels, treatment and recovery paths, and attitudes to life (Cordova et al., 2001; Barskova and Oesterreich, 2009; Bostock et al., 2009; Calhoun and Tedeschi, 2014; Andysz et al., 2015; Ogińska-Bulik, 2015; Rzeszutek et al., 2016; Juczyński and Ogrodnik, 2017). PTG may be affected by gender (but not always) (Tedeschi and Calhoun, 1996, 2004; Barskova and Oesterreich, 2009; Vishnevsky et al., 2010; Ogińska-Bulik, 2013; Akbar and Witruk, 2016; Baghjari et al., 2017), age (Barskova and Oesterreich, 2009; Prati and Pietrantoni, 2009; Ogińska-Bulik, 2013, 2015; Calhoun and Tedeschi, 2014), and time since the traumatic event (Cordova et al., 2001; Barskova and Oesterreich, 2009; Ogińska-Bulik, 2015; Kobosko, 2016; Rzeszutek et al., 2016).

### Posttraumatic Growth and Coping With Stress

Research interest in PTG has also focused on understanding how it relates to the coping strategies employed. Do each of the strategies support—or not support—the growth of PTG in people who have had various health-related traumatic events? In answering this question, studies have highlighted the importance of certain coping processes and strategies (Barskova and Oesterreich, 2009; Prati and Pietrantoni, 2009; Ogińska-Bulik, 2015). The cause of PTG appears not to be the trauma itself, but the way in which affected people cope with it (Barskova and Oesterreich, 2009; Calhoun and Tedeschi, 2014; Ogińska-Bulik, 2015). The literature already distinguished the approach-oriented coping strategies, which are aimed at taking action to reduce the level of stress, and the avoidance-oriented coping strategies, which act by distancing the person from the source of stress (Roth and Cohen, 1986; Carver, 1997).

The approach-oriented coping category includes strategies that are considered effective in coping with stress and its effects, namely, active coping, planning, acceptance, and positive reframing (Barskova and Oesterreich, 2009; Prati and Pietrantoni, 2009; Ogińska-Bulik, 2015; Byra, 2016, Pérez-San-Gregorio et al., 2017; Rzeszutek et al., 2017). Various studies have seen the development of PTG in people with cancer (Barskova and Oesterreich, 2009; Harding et al., 2014; Ogińska-Bulik, 2015; Baghjari et al., 2017; Boals et al., 2019), liver transplants (Pérez-San-Gregorio et al., 2017), and survivors of stroke (Kelly et al., 2018), all through using problem-focused coping and active coping.

At the same time, an increasing number of scientific papers also identify a role for avoidance-oriented coping strategies in the development of PTG, including coping through denial (Barskova and Oesterreich, 2009; Akbar and Witruk, 2016; Pérez-San-Gregorio et al., 2017; Rzeszutek et al., 2017; Kunz et al., 2018). Paradoxically, perhaps, these strategies appear to have what seem like positive outcomes. The recently published longitudinal study by Kunz et al. (2018) shows that the flexible use of both approach- and avoidance-oriented coping strategies contributes to PTG. However, we believed that denial coping may in fact favor the development, not of constructive PTG, but what has been called “illusory” PTG by the creators of the Janus-face model of PTG (Zoellner and Maercker, 2006). Due to self-reporting, the difficulty is to distinguish between the two, but some researchers claimed to have found actual and illusory PTG (Boals et al., 2019).

Coping strategies beyond the typical approach-oriented and avoidance-oriented coping strategies for stress also play an important role in the development of PTG, notably religious coping (Barskova and Oesterreich, 2009; Prati and Pietrantoni, 2009; Ogińska-Bulik, 2014, 2015; Byra, 2016; García et al., 2017; Kelly et al., 2018) and humor (Barskova and Oesterreich, 2009; Rajandram et al., 2011; Ogińska-Bulik, 2014, 2015; Byra, 2016). Certainly, the role of social support (in the form of seeking instrumental and emotional support) in the development of PTG in people affected by trauma is also an invaluable coping strategy, especially in patients with CID (Cordova et al., 2001; Barskova and Oesterreich, 2009; Prati and Pietrantoni, 2009; Rajandram et al., 2011; Scrignaro et al., 2011; Calhoun and Tedeschi, 2014; Ogińska-Bulik, 2015; Byra, 2016; Baghjari et al., 2017; García et al., 2017; Juczyński and Ogrodnik, 2017; Pérez-San-Gregorio et al., 2017; Kelly et al., 2018).

### Posttraumatic Growth, Coping, and Postlingual Deafness

The traumatic event in the spotlight of this work is profound or total loss of hearing. Most postlingually deaf CI users, when confronted with stress as a result of deafness, generally resort to approach-oriented coping strategies, but younger users are more likely to use active coping (Kobosko et al., 2015). Our previous studies have shown that CI users are also likely to resort to avoidance-oriented coping strategies such as self-distraction, denial, venting, and behavioral disengagement; some may use religious coping (Kobosko et al., 2012, 2015). Over the several years of using a CI, the same strategy (e.g., venting) may perform different psychological functions: in patients with a short CI experience venting is negatively correlated with CI satisfaction, but in those with longer CI experience venting correlates positively with CI satisfaction (Kobosko et al., 2015). There are also sex differences; although both postlingually deaf men and women with a CI do not differ overall in coping strategies, women with a CI are more likely to use maladaptive coping strategies as compared with women in the general population (Kobosko et al., 2012).

### This Study

The rationale for this study rests on noting that acquired deafness (and CI implantation as the avenue for treating it) involves not only rehabilitating hearing, speech, and communication, but also engaging dynamic psychological processes. These processes may be social relationships, identity reformulation, recognition of a new reality (either external or internal) related to the acquired disability of an individual, and a search for ways to cope with the situation of no longer being a hearing person. But, to date, there has been no research on the incidence of PTG as a result of acquired deafness and its psychological correlates, prompting us to investigate this phenomenon. We also wanted to look at the relationship between PTG and coping strategies in people with postlingual deafness.

The purpose of this study was to examine postlingually deaf CI users and see whether the PTG phenomenon occurred and, if so, at what level (low, medium, or high). In a similar manner, we wanted to investigate the links between the stress-coping strategies such people use and the level of PTG they attained. Relevant here are the sociodemographic characteristics of the postlingually deaf CI users (i.e., age, gender, education, marital or partnership status, and employment status) and the factors directly linked to deafness (i.e., the years of having serious hearing difficulties or being deaf) and the CI implantation itself (i.e., age at CI, duration of CI use, and satisfaction with their CI).

## Materials and Methods

To assess PTG in postlingually deaf adult CI users, the PTG inventory (PTGI) suggested by Tedeschi and Calhoun (1996) was used. This questionnaire is commonly used to study PTG in people suffering from chronic, severe, or life-threatening illnesses and disabilities. PTGI is relatively short and has good psychometric properties, both in the original version (Tedeschi and Calhoun, 1996) and in its Polish adaptation (Juczyński and Ogińska-Bulik, 2010). For similar reasons, Brief COPE (Carver, 1997) in its Polish adaptation (Juczyński and Ogińska-Bulik, 2009) was chosen to evaluate stress-coping strategies. Its widespread use makes it possible to compare results for both Poland and internationally.

### Study Participants and Procedure

The study included adults with postlingual deafness, i.e., acquired after acquisition of speech and language. The group consisted of *n* = 119 persons (69 women and 50 men) with profound hearing loss (pure tone audiometry showing loss of >90 dB HL at frequencies of 0.25–8 kHz) acquired after age 4. Their ages ranged from 24 to 80 years. Among the 119 participants, 50 also used a hearing aid (HA) in their second ear, whereas 20 had a second CI. The period of first CI use ranged from 0.5 to 24 years, and the length of having serious hearing difficulties or being deaf ranged from 1 to 64 years. The respondents themselves reported the year of hearing loss, and when they had a problem with giving the precise year, then, in accordance with instructions, they gave an indicative number of years from when, in their own opinion, they had a serious hearing problem or deafness. All participants of this study communicated in spoken Polish. Table 1 lists information about the study group: their personal details (i.e., gender, age, education, marital/partnership status, and employment/study status); details of their deafness (i.e., time span of experiencing deafness); and details of their CI (i.e., years of use; age at receiving the CI, satisfaction with the CI, and amplification type). Intergroup comparisons (χ^2^) of the variables in relation to gender were calculated, and there were no significant differences.

**Table 1:** Personal characteristics of subjects, CI data, and hearing status of all participants.

		**All participants**	**Men**	**Women**	**Statistical test between men/women**
*N*		119	50	69	
Age, yr	Mean (SD)	57 (12)	59 (12)	57 (13)	*t*_(117)_ = 0.651; *NS*
	Range	24–80	27–79	24–80	
*N* (younger <60 yr)		60	24	36	χ^2^(1) = 0.202; *NS*
*N* (older >60 yr)		59	26	33	
Educational status	Primary or secondary (%)	62.2	64.0	60.9	χ^2^(1) = 0.121; *NS*
	Diploma or university (%)	37.8	36.0	39.1	
Marital (partnership) status	In a relationship (%)	32.8	24.0	39.1	χ^2^(1) = 3.012; *NS*
	Not in a relationship (%)	67.2	76.0	60.9	
Employment (or study)	Employed (or studying) (%)	45.4	48.0	43.5	χ^2^(1) = 0.239; *NS*
	Unemployed (%)	54.6	52.0	56.5	
Duration of deafness (yr)	Mean (SD)	26 (15)	24 (17)	28 (14)	*t*_(117)_ = −1.14; *NS*
	Range	1–64	1–64	2–60	
Age at CI	Mean (SD)	51 (13)	52 (14)	51 (13)	*t*_(117)_ = 0.204; *NS*
	Range	14–78	14–78	22–75	
CI experience	Mean (SD)	6 (5)	7 (6)	6 (4)	*U* = 1,645.5; *NS*
	Range	0.5–24	0.5–24	0.5–21	
CI satisfaction (%)	Mean (SD)	81 (24)	80 (25)	82 (22)	*U* = 1,707; *NS*
	Range	0–100	0–100	0–100	
Type of amplification	CI (%)	40.3	50.0	33.3	χ^2^(2) = 4.062; *NS*
	CI+HA (%)	42.0	32.0	49.3	
	2 CI (%)	17.7	18.0	17.4	

*NS, not significant (P > 0.05)*.

The subjects were recruited from CI patients of the Institute of Physiology and Pathology of Hearing. During a rehabilitation camp, they were given an envelope with a cover letter asking them to voluntarily and anonymously participate in a scientific research project. The envelope also contained questionnaires that could be returned in a sealed envelope to the camp supervisor. The response rate was around 50%. This is slightly less than in other studies conducted in our center by the correspondence method (Kobosko et al., 2015). The lower rate may be because some people felt uncomfortable in handing the completed questionnaires to the camp supervisor. As the data were collected without identifying the patients, it was not possible to match the questionnaires with audiometric tests; however, other studies have shown (de Graaf and Bijl, 2002; Fellinger et al., 2012; Kobosko et al., 2015) that there is no correlation between audiological outcomes and psychological well-being. Therefore, in this study, the priority was to obtain the most honest responses from the patients, which can only be done anonymously.

Statistical analysis was performed using the IBM SPSS software program, version 25.0 (IBM Corp., Armonk, NY, USA) and its following tests: Pearson's χ^2^ (for assessing differences between sociodemographic data), Student's *t*-test (for assessing differences between descriptive statistics of the psychological questionnaires), Pearson's correlation coefficient (for correlating PTG to psychological variables and the personal data of patients), and linear multiple regression (for assessing which variables can be used as predictors of PTG). A 95% confidence level (*P* < 0.05) was chosen as the criterion of significance. When conducting multiple comparisons, *P*-values were adjusted using the Benjamini–Hochberg procedure to control for false discovery rates (Benjamini and Hochberg, 1995).

All procedures were approved by the ethics committee of the Institute of Physiology and Pathology of Hearing, Warsaw (approval number IFPS: /KB/02/2014). No written consent was provided, as the study was based on anonymous questionnaires.

### Measures

*Post-traumatic Growth Inventory (PTGI)*. This instrument (Tedeschi and Calhoun, 1996; Juczyński and Ogińska-Bulik, 2010) was used to assess positive changes flowing from the experience of acquired deafness. PTGI consists of 21 items, each of which has possible answers ranging from “0” (*I did not experience this change as a result of my crisis*) to “5” (*I experienced this change to a very great degree as a result of my crisis*). Instructions made it clear that answers should relate to one event, i.e., the loss of hearing. Examples of items from PTGI include *I can better appreciate each day*, or *I discovered that I'm stronger than I thought I was*. In the Polish adaptation, factor analysis resulted in an inventory consisting of four dimensions (and not, as in the original, 5). Factor analysis measured positive changes in the following areas: changes in perception of oneself, changes in relationships with others, greater appreciation for life, and spiritual changes. The sum of all these items gave a global PTGI, which ranged from 0 to 105 points. The higher the score, the more positive are the changes resulting from the traumatic event. According to Juczyński and Ogińska-Bulik (2009), Cronbach's α for the Polish version of PTGI is 0.93, and for the four dimensions, it varies from 0.63 to 0.87. In this study, Cronbach's α for PTGI was found to be 0.95, and for the individual dimensions it was 0.70–0.92.

*Brief Coping Orientation to Problems Experienced (Brief COPE)*. This self-reporting questionnaire (Carver, 1997; Juczyński and Ogińska-Bulik, 2009) contains 28 items that assess coping strategies. It uses a 4-point scale from “0” (*I never do this*) to “3” (*I almost always do this*). Brief COPE measures 14 separate strategies for coping with stress based on a 2-item subscale. The strategies are as follows: *active coping* (undertaking actions aimed at improving the situation); *planning* (thinking and planning what to do); *positive reframing* (perceiving the situation in a better light); *acceptance* (accepting the situation and learning how to live with it); *humor* (joking and treating the situation as a game); *turning to religion* (praying and meditating to find consolation); *using emotional support* (seeking reassurance, understanding, and the support of other people); *using instrumental support* (seeking advice and aid from other people), *self-distraction* (taking up other activities to avoid thinking about the event); *denial* (denying the existence of the situation); *venting* (displaying negative emotions); *substance use* (resorting to psychoactive substances, e.g., alcohol, to alleviate unpleasant emotions); *behavioral disengagement* (hopelessness, giving up attempts to attain the goals of an individual); and *self-blame* (criticizing and blaming oneself for what happened). Each strategy is evaluated on the basis of the average of the two items. Reliability of Brief COPE was calculated using a split-half measure and the Guttman coefficient was 0.81.

The *inquiry form* included questions about personal characteristics (i.e., gender, age, education, marital or partner status, employment, or study status), as well as variables related to deafness, e.g., *when the loss of hearing happened*, and questions related to the CI, e.g., *how long have you been using the CI*. There was one question to measure the satisfaction of subjects with their CI. The subject marked on a line the degree to which they were pleased with their CI: the line had no visible markings except it had a 0 at one end (dissatisfied) and a 10 at the other (very satisfied). The line, 161-mm long, was placed on a single page; the response was measured in millimeters and converted to a percentage. More about the method used for measuring CI satisfaction can be found elsewhere (Kobosko et al., 2015).

## Results

### Descriptive Statistics

First the global levels of PTG for each subject were evaluated and referenced to the Polish sten standards derived from people who have had various other sorts of traumatic events. A score for global PTG of 0–53 points was rated as low PTG (stens 1–4), a score of 54–72 was rated as average PTG (stens 5 and 6), and a score of 73–105 (stens 7–10) was rated as high PTG (Juczyński and Ogińska-Bulik, 2010). The postlingually deaf CI users in this study had a global level of PTG of about average as compared with Polish standards; 31% had high global PTG values and 32% had average global PTG.

Table 2 shows the results of PTGI (for four dimensions, and globally) for all participants, as well as divided by gender. No significant differences were observed between men and women.

**Table 2:** Posttraumatic Growth Inventory (PTGI) values for all participants, divided by gender.

**Psychological variables**	**Mean (SD)**	**Mean (SD)**	**Mean (SD)**	**Comparison between men and women**
	**All participants**	**Men**	**Women**	
Global posttraumatic growth (PTG)	61.36 (22.75)	58.27 (23.20)	63.70 (22.35)	*t*_(117)_ = −1.140; *P* = 0.42; *d* = 0.23
Changes in perception of oneself	25.85 (10.48)	25.38 (10.74)	26.18 (10.36)	*t*_(117)_ = −0.382; *P* = 0.70; *d* = 0.07
Changes in relationships with others	21.97 (7.47)	21.45 (7.79)	22.34 (7.28)	*t*_(117)_ = −0.630; *P* = 0.66; *d* = 0.11
Greater appreciation for life	9.46 (3.83)	8.73 (3.98)	10.00 (3.66)	*t*_(117)_ = −1.772; *P* = 0.20; *d* = 0.33
Spiritual changes	4.83 (3.27)	4.10 (3.34)	5.41 (3.12)	*t*_(117)_ = −2.100; *P* = 0.19; *d* = 0.40

*Results are shown for four dimensions (rows) as well as globally. Results of statistical tests for difference between gender groups are provided in the last column (test, significance level, Cohen's d). There were no statistically significant differences between men and women*.

Table 3 shows results of the Brief COPE inventory, again for all participants and divided by gender. Men and women differed in turning to religion, using emotional support, using instrumental support, self-distraction, denial, and venting. In these strategies, women had higher scores and the effect sizes were medium to large (0.5–1.0).

**Table 3:** Results of Brief COPE questionnaire for all participants, divided by gender.

**Coping strategy**	**Mean (SD)**	**Mean (SD)**	**Mean (SD)**	**Comparison between men and women**
	**All participants**	**Men**	**Women**	
Active coping	2.21 (0.71)	2.18 (0.86)	2.22 (0.58)	*t*_(117)_ = −0.201; *P* = 0.90; *d* = 0.05
Planning	2.19 (0.67)	2.14 (0.81)	2.23 (0.55)	*t*_(117)_ = −0.736; *P* = 0.53; *d* = 0.13
Positive reframing	1.87 (0.65)	1.93 (0.70)	1.83 (0.61)	*t*_(117)_ = 0.865; *P* = 0.49; *d* = 0.15
Acceptance	2.22 (0.57)	2.29 (0.62)	2.16 (0.54)	*t*_(117)_ = 2.259; *P* = 0.38; *d* = 0.22
Humor	0.88 (0.73)	0.89 (0.72)	0.88 (0.74)	*t*_(117)_ = 0.097; *P* = 0.92; *d* = 0.01
Religion	1.34 (1.09)	0.94 (1.10)	1.64 (0.98)	*t*_(117)_ = −3.631; *P* = 0.0005^***^; *d* = 0.67
Use of emotional support	1.68 (0.73)	1.47 (0.78)	1.83 (0.66)	*t*_(117)_ = −2.742; *P* = 0.016^*^; *d* = 0.50
Use of instrumental support	1.71 (0.64)	1.50 (0.68)	1.86 (0.56)	*t*_(117)_ = −3.183; *P* = 0.006^**^; *d* = 0.80
Self-distraction	1.67 (0.80)	1.29 (0.84)	1.94 (0.64)	*t*_(117)_ = −4.773; *P* = 0.0005^***^; *d* = 0.87
Denial	0.81 (0.78)	0.55 (0.69)	1.00 (0.81)	*t*_(117)_ = −3.179; *P* = 0.006^**^; *d* = 0.60
Venting	1.19 (0.67)	0.84 (0.57)	1.44 (0.63)	*t*_(117)_ = −5.379; *P* = 0.0005^***^; *d* = 1.00
Substance use	0.21 (0.45)	0.32 (0.55)	0.14 (0.35)	*t*_(117)_ = −3.179; *P* = 0.058; *d* = 0.39
Behavioral disengagement	0.68 (0.57)	0.55 (0.52)	0.77 (0.58)	*t*_(117)_ = −2.161; *P* = 0.058; *d* = 0.40
Self-blame	1.11 (0.74)	1.04 (0.84)	1.16 (0.66)	*t*_(117)_ = −0.920; *P* = 0.49; *d* = 0.16

*Results of statistical tests for differences between gender groups are listed in the last column (test, significance level, Cohen's d)*.

* *P < 0.05;*

** *P < 0.01;*

*** *P < 0.001*.

### Correlations

Table 4 shows Pearson's correlations (for four dimensions and for global values) for the deafness experience of participants, duration of CI experience, and strategies for coping with stress (from Brief COPE). Age, age at CI, and CI satisfaction were not correlated with PTG. Duration of deafness correlated with changes in perception of oneself, changes in relationships with others, and global PTG (for men and for the whole group). CI experience correlated with changes in perception of oneself, greater appreciation for life (for women and for the whole group), and with global PTG for the whole group.

**Table 4:** Pearson's *r* correlations of age, deafness experience, age at CI, years of CI experience, CI satisfaction, and coping strategies (Brief COPE) of participants with posttraumatic growth (for four dimensions and for global PTGI) among all participants, and divided by gender.

	**Group**	**Global posttraumatic growth**	**Changes in perception of oneself**	**Changes in relationships with others**	**Greater appreciation for life**	**Spiritual changes**
Duration of deafness	Men	0.35^*^	0.32^*^	0.34^*^	NS	NS
	Women	NS	NS	NS	NS	NS
	All	0.23^*^	0.21^*^	0.20^*^	NS	NS
CI experience	Men	NS	NS	NS	NS	NS
	Women	NS	0.27^*^	NS	0.27^*^	NS
	All	0.21^*^	0.26^**^	NS	0.25^**^	NS
Active coping	Men	NS	NS	NS	NS	NS
	Women	0.38^**^	0.32^*^	NS	NS	NS
	All	NS	NS	NS	NS	NS
Planning	Men	NS	NS	NS	NS	NS
	Women	0.36^**^	0.37^**^	NS	NS	NS
	All	NS	NS	NS	NS	NS
Acceptance	Men	NS	NS	NS	NS	NS
	Women	NS	NS	0.28^*^	NS	NS
	All	NS	NS	NS	NS	NS
Religion	Men	NS	NS	NS	NS	0.58^*^
	Women	NS	NS	NS	NS	0.55^***^
	All	0.23^*^	NS	0.22^*^	NS	0.59^**^
Use of emotional support	Men	NS	NS	NS	NS	NS
	Women	0.28^*^	NS	0.28^*^	NS	NS
	All	NS	NS	0.29^**^	NS	NS
Use of instrumental support	Men	NS	NS	0.43^**^	NS	NS
	Women	0.34^*^	NS	0.39^**^	NS	0.39^**^
	All	0.33^**^	0.19^*^	0.41^**^	NS	0.30^**^
Self-distraction	Men	NS	NS	NS	NS	NS
	Women	0.32^*^	0.35^**^	0.25^*^	NS	NS
	All	0.24^*^	0.25^*^	0.20^*^	0.26^**^	NS
Denial	Men	NS	NS	NS	NS	NS
	Women	0.31^*^	NS	0.26^*^	NS	0.31^*^
	All	0.28^**^	NS	0.19^*^	0.18^*^	NS
Venting	Men	NS	NS	NS	NS	NS
	Women	0.31^*^	0.30^*^	NS	NS	NS
	All	0.27^**^	0.21^*^	0.20^*^	0.19^*^	0.20^*^
Behavioral disengagement	Men	NS	NS	NS	NS	NS
	Women	NS	NS	NS	NS	NS
	All	NS	NS	NS	NS	0.26^**^

*Only variables for which there were significant correlations are shown*.

*NS, not significant*.

* *P < 0.05;*

** *P < 0.01;*

*** *P < 0.001*.

*Significant correlations are shown in bold*.

On the contrary, the Brief COPE correlated with PTG in several respects. In the case of the whole group, global PTG correlated significantly with religion, use of instrumental support, self-distraction, denial, and venting. In terms of all the dimensions of PTG, changes of perception of oneself correlated with the use of instrumental support, self-distraction, and venting. Changes in relationships with others correlated with religion, use of emotional support, use of instrumental support, self-distraction, denial, and venting. Greater appreciation for life correlated with self-distraction, denial, and venting. Finally, spiritual changes correlated with religion, use of instrumental support, venting, and behavioral disengagement.

There were some differences between the genders. Here, we focused on correlations that did not show up as significant for the whole group but were significant for one or the other gender. Hence, in the case of global PTG, men did not show any significant correlations. When analyzing correlations of Brief COPE to dimensions of PTG, for men changes in relationships with others correlated with the use of instrumental support, and spiritual changes correlated with coping using religion. For women, in the case of global PTG there were significant correlations with active coping, planning, and use of emotional support. For dimensions of PTG, women showed significant correlations between changes in perception of oneself and active coping and with planning. For changes in relationships with others, there were significant correlations with acceptance and use of emotional support. For spiritual changes, there were significant correlations with religion and denial.

### Regression Analysis

Regression analysis was performed for PTGI and all personal characteristics and variables related to deafness and CI—age at CI, deafness experience, CI experience, and CI satisfaction—and the results are shown in Table 5. In the case of global PTG, the predictors were partnership status and education. People who were in a relationship had higher global PTG. On the contrary, people with lower education had higher global PTG.

**Table 5:** Stepwise linear regression analysis for all participants.

	**B**	**SE**	**β**	***t***
**Global posttraumatic growth**				
Partnership status	−9.267	4.331	−0.215^*^	−2.140
Education	−12.659	4.086	−0.312^**^	−3.098
**Changes in perception of oneself**				
CI experience (years)	0.441	0.130	0.317^**^	3.398
**Changes in relationship with others**				
Education	−2.872	1.081	−0.251^**^	−2.656
**Greater appreciation for life**				
Gender	1.468	0.699	0.190^*^	2.102
CI experience (years)	0.183	0.067	0.248^**^	2.740
**Spiritual changes**				
Gender	1.703	0.558	0.269^**^	3.052
Education	−2.682	0.573	−0.413^***^	−4.681

*Gender, sociodemographics, CI and hearing status as predictors of posttraumatic growth (PTGI). Only significant variables are shown*.

* *P < 0.05;*

** *P < 0.01;*

*** *P < 0.001*.

The only significant predictor of changes in perception of oneself was CI experience—the longer the better. The only significant predictor of changes in relationship with others was education—again the lower the better. The predictors of greater appreciation for life were gender (better for women) and also CI experience (the longer the better). Predictors of spiritual changes were gender (better for women) and also education (again the lower the better).

Finally, regression analysis was performed, for global PTG and the four PTG dimensions, using coping strategies (Brief COPE results) as predictors (Table 6). For all participants, there were two strong predictors of global PTG, namely, the use of instrumental support and denial. However, for men there was only one predictor, i.e., denial, whereas for women there were three predictors, i.e., active coping, use of emotional support, and denial.

**Table 6:** Stepwise linear regression analysis for all participants and according to gender.

	**All participants**			
	**B**	**SE**	**β**	***t***
**Global posttraumatic growth**				
Use of instrumental support	10.185	3.469	0.293^**^	2.936
Denial	7.904	2.415	0.326^**^	3.272
**Changes in perception of oneself**				
Self-distraction	4.452	1.209	0.357^***^	3.682
**Changes in relationship with others**				
Use of instrumental support	4.052	1.081	0.368^***^	3.748
Denial	2.114	0.737	0.282^**^	2.870
**Greater appreciation for life**				
Self-distraction	1.443	0.390	0.340^***^	3.699
**Spiritual changes**				
Planning	−0.809	0.359	−0.164^*^	−2.255
Religion	1.842	0.217	0.633^***^	8.500
Use of instrumental support	1.013	0.385	0.197^*^	2.632
	**Men**			
	**B**	**SE**	***β***	***t***
**Global posttraumatic growth**				
Denial	11.566	5.030	0.354^*^	2.299
**Changes in perception of oneself**				
Denial	7.241	2.865	0.371^*^	2.528
**Changes in relationship with others**				
Active coping	−3.537	1.267	−0.378^**^	−2.792
Use of instrumental support	6.456	1.516	0.576^***^	4.260
**Greater appreciation for life**				
Planning	−1.428	0.629	−0.294^*^	−2.269
Self-distraction	2.040	0.589	0.449^**^	3.463
**Spiritual changes**				
Planning	−1.564	0.456	−0.390^**^	−3.427
Religion	1.843	0.349	0.600^***^	5.280
	**Women**			
	**B**	**SE**	***β***	***t***
**Global posttraumatic growth**				
Active coping	20.841	3.857	0.568^***^	5.404
Use of emotional support	8.146	3.062	0.278^*^	2.660
Denial	8.397	2.322	0.378^**^	3.615
**Changes in perception of oneself**				
Planning	7.894	2.190	0.409^**^	3.605
Self-distraction	5.954	1.654	0.409^**^	3.599
**Changes in relationship with others**				
Active coping	2.414	1.074	0.253^*^	2.247
Acceptance	2.898	1.044	0.300^**^	2.777
Use of instrumental support	3.115	1.011	0.332^**^	3.079
Denial	1.606	0.699	0.255^*^	2.296
Self-blame	−1.973	0.884	−0.253^**^	−2.233
**Spiritual changes**				
Religion	1.849	0.278	0.647^***^	6.640
Use of instrumental support	1.493	0.510	0.285^**^	2.929

*Coping strategies (Brief COPE) as predictors of posttraumatic growth (PTGI). Only significant variables are shown*.

* *P < 0.05;*

** *P < 0.01;*

*** *P < 0.001*.

When coping strategies (Brief COPE) were used as predictors of changes of perception of oneself, the main predictor for the group as a whole was self-distraction. However, for men it was denial, whereas in the female subgroup there were also planning and self-distraction. For the whole group the predictors of changes in relationships with others were use of instrumental support and denial. For men, the predictors were active coping and instrumental support, whereas for women there were in addition acceptance, denial, and self-blame. As predictors of greater appreciation for life, Brief COPE revealed self-distraction for the whole group and, for men, planning as well; however, for women, there were no significant predictors at all. Finally, when looking for predictors of spiritual changes, the significant coping strategies were planning, religion, and use of instrumental support. Planning and religion were significant predictors among men, whereas religion and instrumental support were significant predictors among women.

## Discussion

This study has attempted to measure the occurrence of PTG in individuals who have experienced profound (or total) postlingually acquired hearing loss and are currently using a CI. We also investigated the links between PTG level and sociodemographic characteristics, as well as variables related to deafness and CI, but above all with coping strategies. To the best of our knowledge, these are the first studies on the occurrence of PTG, and its relation to coping strategies in CI users (looking also at the role of gender).

### Posttraumatic Growth in Postlingually Deaf CI Users

The results indicate that, like other trauma sufferers, postlingually deaf CI users undergo PTG and reach a similar level to those who have had spinal cord injury (Byra, 2016), AIDS/HIV diagnosis (Rzeszutek et al., 2016; Rzeszutek and Gruszczyńska, 2018), breast cancer (Cordova et al., 2001; Koutrouli et al., 2014; Andysz et al., 2015), or serious heart disease (Bluvstein et al., 2013). Referring to Polish sten norms for trauma sufferers (Juczyński and Ogińska-Bulik, 2010), it can be said that 31% of postlingually deaf CI users experienced a high level of PTG and 32% experienced a medium level. In total, this means we should expect that about two-thirds of our study participants went through PTG following their postlingual profound hearing loss or deafness.

### PTG and Sociodemographic Characteristics

From a meta-analysis of research into PTG, it appears that the female gender is associated with a higher PTG in response to stressful events (Barskova and Oesterreich, 2009; Vishnevsky et al., 2010; Ogińska-Bulik, 2015; Akbar and Witruk, 2016; Rzeszutek and Gruszczyńska, 2018; Wu et al., 2019). In our study, female gender proved to be a significant predictor in two dimensions of PTG, namely, the spiritual changes domain and greater appreciation for life. Other research also indicates the important role of gender in experiencing PTG in the area of spiritual changes (Steel et al., 2008; Cormio et al., 2015; Rzeszutek et al., 2016).

It turned out, however, that lower education was a good predictor of PTG—not just for global PTG but also for positive changes in the spiritual domain and in relationships with others. Similar results between PTG and lower education have been obtained by a few other researchers in relation to women with breast cancer (Cordova et al., 2001; Koutrouli et al., 2014), in long-term colorectal cancer survivors (Jansen et al., 2011), in persons after bone marrow transplantation for cancer (Widows et al., 2005), in patients with coronary artery disease (Leung et al., 2012), and in patients in the years after lung transplantation (Fox et al., 2014), as well as in women after induced abortion (Barraza Illanes and Calvo-Francés, 2018).

Our research shows that lower education contributes to the development of PTG. Perhaps, as other researchers have pointed out, people with lower education (and thus of generally lower socio-economic status) experience more hardship in their lives and so have more experience in finding the positive side of highly stressful life events (Ross and Van Willigen, 1997; Jansen et al., 2011). It is also possible that less educated people have lower expectations in various spheres of life, including in relation to other people, which may be associated with feeling less dissatisfied than do more educated persons (Ross and Van Willigen, 1997), and in consequence they experience higher PTG. However, the direction of the relationship between PTG and the level of education requires further research.

Among the sociodemographic variables, marital or partnership status proved to be very important, and hence the relationship between the spouse and the partner seems to favor the development of PTG. This points to the interpersonal aspect of PTG: the creators of the PTG concept, Tedeschi and Calhoun, themselves indicate the importance of relations with people and of social support (Calhoun and Tedeschi, 2014), findings similar to those of other PTG studies (Barskova and Oesterreich, 2009; Harding et al., 2014; Ogińska-Bulik, 2015; Rzeszutek and Gruszczyńska, 2018; Sörensen et al., 2019). Other sociodemographic characteristics such as age or employment status did not show a link with PTG.

### PTG and Deafness-Related Factors

The duration of deafness correlated with positive changes after the trauma, especially in the dimensions of changes in perception of oneself and changes in relationships with others, although this relationship applied only to men. The creators of the PTG concept, Tedeschi and Calhoun, believed that the time elapsed from trauma is conducive to experiencing positive changes (Calhoun and Tedeschi, 2014), but the results of research on the relationship between the elapsed time and PTG are inconclusive (Barskova and Oesterreich, 2009; Koutrouli et al., 2014; Ogińska-Bulik, 2015; Rzeszutek and Gruszczyńska, 2018). Some others pointed to similar positive relationships, as found in the case of long-term disease-free cancer survivors (Cormio et al., 2015), women with breast cancer (Cordova et al., 2001), or mothers of deaf children experiencing PTG associated with the diagnosis of deafness in their child (Kobosko, 2016). However, it should be noted that in the few studies reporting PTG, there was always a long period of coping with CID, as was the case in our study.

The duration of CI experience turned out to be a predictor of PTG in postlingually deaf CI users in relation to two PTG dimensions, namely, changes in perception of oneself and greater appreciation of life. This result indirectly shows that a CI is highly important to users, a conclusion often supported by the CI users themselves, who, especially in qualitative research, report the enormous benefits associated with a CI not only in the hearing-related sphere but also in many spheres of life (Hallberg and Ringdahl, 2004; Heydebrand et al., 2005; Kennedy et al., 2008; Rembar et al., 2009; Ramos-Macías et al., 2016). In our research, we considered the CI to be an object that the person has sought out in response to their hearing loss (i.e., the CI is a part of the coping strategy of users—specifically, it is active coping or coping by instrumental support). A similar positive relationship has been found between the time since a heart transplant and PTG (Juczyński and Ogrodnik, 2017). However, it should be remembered that recovery from some illnesses can be enhanced by PTG and well-being (Calhoun and Tedeschi, 2014; Ogińska-Bulik, 2015; Martz and Livneh, 2016), suggesting that people experiencing PTG are more able to appreciate what cochlear implantation offers them and the “return to the world of sounds.” In future, more longitudinal studies are needed to explore the relationship between PTG and the time since trauma of hearing loss or the time since medical intervention, such as receiving a CI, took place (Wu et al., 2019). Other variables related to deafness—the age at which the CI was received and satisfaction with the device—did not show links with the development of PTG.

### Coping With Stress in Postlingually Deaf CI Users in Relation to Gender

It turned out that postlingually deaf women with a CI, more often than did deaf men with CI, resorted to avoidance-oriented coping strategies when confronted with stress, including self-distraction, denial, and venting, which, in the long run, are mostly regarded as ineffective (Roth and Cohen, 1986; Ottenbreit and Dobson, 2008; Ogińska-Bulik, 2015; Wu et al., 2019). This result may point to higher mental health problems in postlingually deaf women because we know that, for example, in response to stress, there is a connection between avoidance-oriented coping and depressive symptoms (Ottenbreit and Dobson, 2008). The results of other studies indicate that postlingually deaf women, as compared with postlingually deaf men, exhibit a higher level of mental distress, including depression or anxiety (de Graaf and Bijl, 2002; Kvam et al., 2007; Fellinger et al., 2012; Kim et al., 2017). In our study, deaf women with a CI also used strategies related to seeking emotional and instrumental support more often than did men, as well as turning to religion (favoring PTG), which is also indicated by the results we obtained.

### PTG and Coping With Stress in Postlingually Deaf CI Users

The results show that, in postlingually deaf people with CIs, both approach-oriented and avoidance-oriented coping strategies are significantly associated with PTG. In effecting positive changes after the trauma of hearing loss, an important coping strategy in our study group was turning to religion and seeking social support, both of which can be categorized as employing emotional or instrumental support.

#### PTG and Approach- and Avoidance-Oriented Coping Strategies

Among the several approach-oriented coping strategies that foster global PTG, two were active coping and planning, and these strategies were found to have positive predictive power, but only for women. Here, a positive relationship with PTG occurred in the dimensions of self-perception and in global PTG. Only in women did this acceptance-coping strategy play an important role in developing positive changes in relations with other people. In other studies, such as in those with spinal cord injury or cancer patients, effective strategies—such as taking additional action or trying to get rid of the problem—have been strongly associated with PTG (Barskova and Oesterreich, 2009; Scrignaro et al., 2011; Ogińska-Bulik, 2015; Wu et al., 2019). In cases of people with CID, acceptance helps facilitate PTG, as has been found in studies with cancer patients (Prati and Pietrantoni, 2009), liver transplant recipients (Pérez-San-Gregorio et al., 2017), and rheumatoid arthritis patients (Rzeszutek et al., 2017).

In contrast, for postlingually deaf men with CIs, an active coping strategy was not conducive to PTG, especially in the area of relations with other people, where it remained a negative correlation. The coping strategy of planning also played an important role in two areas of PTG in men with postlingual deafness, but as in the case with the active coping strategy, the use of this strategy was associated with a lower level of PTG. This probably happens because activity (i.e., active coping and planning) does not focus on solving the problem; instead, the men were trying to cope emotionally with the situation, and having to deal with negative emotions while adapting to their chronic disability (Livneh, 2009; Kelly et al., 2018).

As far as strategies related to avoidance-oriented coping are concerned, denial remained in a positive relation to global PTG for the whole group of study participants, both men and women, being a predictor in the areas of PTG change in relations with others (women) and changes in perception of oneself (men). Denial, i.e., “I reject the idea this ever happened,” is already known to be a coping strategy that does not generally lead to good health (Livneh, 2009), positive treatment outcomes (e.g., satisfaction with their CI; Kobosko et al., 2015), or successful adaptation to the acquired disability (Livneh, 2009). Nevertheless, recent studies indicate that the use of this strategy can promote PTG, and hence play a positive role in psychological adaptation to CID. For example, PTG emanating from denial has been seen in liver transplant recipients (Pérez-San-Gregorio et al., 2017), people with spinal cord injury (Kunz et al., 2018), stroke survivors (Kelly et al., 2018), and earthquake survivors (Akbar and Witruk, 2016). However, the positive or negative effect of denial will vary, depending (among other things) on the stage of adaptation to the highly stressful life event, the time since it occurred, and its context.

Kunz et al. (2018) suggested an explanation that points to the flexible use of different types of coping strategies, i.e., use of both approach- and avoidance-oriented strategies (coping flexibility) facilitates PTG, and this will of course be available to people with postlingual deafness. It might also be presumed that the use of denial serves to reduce distress, and hence facilitates PTG, as other researchers have suggested (Kelly et al., 2018). It should be acknowledged, however, that in the case of some postlingually deaf subjects with a CI, the PTG may be illusory, as in the Janus-face model of PTG (Zoellner and Maercker, 2006). This question still requires further research, perhaps by using clinical psychological diagnosis of survivors of trauma, including hearing loss.

Self-distraction (i.e., avoiding thinking about the consequences of a stressful life event by engaging in other activities such as sleep, binge eating, or watching TV) is another coping strategy classed as avoidance-oriented. We found that it actually favors PTG in postlingually deaf people with a CI and was a predictor of PTG change in the perception of oneself (in women), and in the sphere of greater appreciation of life (in men). Its role in the development of PTG is similar to that of others in this group, i.e., it favors reduction of mental distress (Akbar and Witruk, 2016; Kelly et al., 2018).

#### PTG and Use of Social Support

Social support is a psychosocial resource that, according to the model of Calhoun and Tedeschi (2014), promotes the development of PTG through activating cognitive processing of trauma, e.g., by finding the meaning of the traumatic event. The present results are consistent with other work on the subject, but point to a special role for the use of instrumental support. Although coping using this strategy worked for all study participants, it was stronger for women, where it turned out to be a predictor. For women, use of instrumental support was important for positive changes in relationships with others. Only for women who used this form of support did it predict positive PTG in the dimension of spiritual change.

For postlingually deaf adults with a CI, it is particularly important to provide social support in the form of specific help, such as CI information and advice (included here in our definition of instrumental support coping). Help can take the form of support from a team of specialists—physician, speech therapist, psychologist, teacher, audiologist, or clinical engineer—who are called on when qualifying for a CI, during surgery, or later (during hearing and speech rehabilitation).

For all participants, the use of emotional support turned out to be a coping strategy well-correlated with changes in relationships with others, and, for women, with the development of global PTG and changes in relationships with others. This finding is consistent with the results of research on PTG in women with breast cancer (Barskova and Oesterreich, 2009; Koutrouli et al., 2014; Ogińska-Bulik, 2015). This result might be explained by a tendency for women to have more social contact and acceptance, particularly in Middle Eastern and European cultures, when reaching out for help and support (Akbar and Witruk, 2016; Sörensen et al., 2019). Familiar people, family, and friends, as well as other postlingually deaf CI patients in a similar situation, can be a source of enormous social, emotional, and instrumental support. This is especially true for significant others (i.e., partners), so, according to our results with postlingually deaf CI users, being in a relationship will support PTG.

#### PTG and Turning to Religion

Postlingually deaf people with a CI, especially women, more often reach for religion to cope than do people from the general population (Kobosko et al., 2012). In the cultures of Central and Eastern Europe, religion is a major source of support, especially during illness, cataclysm, and misfortune (Stecz and Kocur, 2015). For postlingually deaf participants with CIs, our results show that a religious coping strategy provides a positive, though weak, relationship with the development of global PTG; we found a correlation of PTG in the dimensions of change in relationships with others and with the domain of spiritual changes. As a predictor, religion turned out to be significant only for spiritual changes (in both women and men).

### Limitations and Ideas for Future Studies

It would be interesting to conduct detailed longitudinal studies in this area, looking at PTG at various time intervals beginning from the incidence of hearing loss. The role of cochlear implantation in this process is still unexplored. In this study, we treated the CI as one manifestation of invoked coping strategies, in this way having an indirect effect on PTG. However, due to the widespread availability of CIs in many countries, it is currently difficult to find a control group of postlingually deaf people who do not use this hearing prosthesis.

In addition, since the responses of participants were anonymous, in this study it was not possible to correlate PTG results with objective benefits from using a CI. Subjective assessment of the CI, measured as CI satisfaction, was found not to be related to PTG. In further studies on PTG, it would be worth considering not only satisfaction with a CI but also prior expectations about receiving a CI, and how these relate to its objective benefits. How PTG depends on these factors might help improve rehabilitation after receiving a CI, and also tell us more about the general psychosocial and hearing-related functioning of CI users.

It might also be helpful to collect the perspectives of family members and others close to the postlingually deaf person. In future studies, two groups of people with postlingual deafness and CIs might also be compared, i.e., those whose hearing deteriorated gradually and those who experienced sudden deafness.

Since we used a single PTGI questionnaire, this set of self-reported data could not be considered robust. Self-reported measures, in which “growth” changes are declared by the respondent, mean the answers might just reflect psychological defense mechanisms and that no real change actually occurred.

A particular limitation is the study group itself: it was heterogeneous due to the time that had elapsed since hearing loss. This factor is difficult to control when hearing loss occurs gradually; many of the subjects were then unable to say exactly how long ago they had lost their hearing. This time variable may be important, as cochlear implantation is just one of the actions/behaviors undertaken by postlingually deaf people to cope with their condition. Another limitation of the study is that the patient cohort was relatively small for thematic analysis, given the disparate nature of CI users.

This study used participants who agreed to fill in questionnaires, and in future it would be informative to use other approaches to gauging PTG, e.g., by using one-on-one clinical interviews. Although our study consistently found that coping strategies were related to PTG, the cross-cultural differences in this area cannot be ruled out.

## Conclusion

In general, about two-third of postlingually deaf CI users in our study experienced PTG at a high or medium level. We found that the length of time since hearing was lost favors the development of PTG, especially in men. Also, the time elapsed since the CI operation—which we identify as the manifestation of an active coping strategy or of seeking instrumental support—turned out to work in favor of PTG.

We found in our study participants that a higher level of global PTG was associated with certain stress-coping strategies: for women mainly active coping, planning, use of emotional support, and denial; for men, denial, and, for all postlingually deaf CI users: use of instrumental support and denial. That is, both approach- and avoidance-oriented coping strategies are used in the development of global PTG.

An important issue is the availability of professional support. Practitioners need to be aware of PTG arising as a natural result of a highly stressful life event (but not in everybody)—severe and profound hearing loss in postlingually deaf CI users. This group of patients should be made aware of the positive role of PTG in adapting to their deafness, as well its role in improving hearing and speech rehabilitation. A parallel can be drawn to other clinical groups (say coronary disease outpatients) where it has been shown that there is a positive correlation between PTG and health service use—that is, greater PTG is connected with higher levels of rehabilitation program enrollment (Leung et al., 2012).

We believe that the various forms of psychological interventions and psychoeducation offered to postlingually deaf people with a CI should be targeted to those stress-coping strategies which favor PTG. However, it would be best to offer psychological interventions during the initial audiological rehabilitation—that is, psycho-education, psychotherapy, training in how to cope with the stress associated with acquired deafness, and self-help programs (Garnefski and Kraaij, 2012). As we now know, psychosocial adaptation to deafness and PTG are directly and positively related, and therefore are mutually beneficial.

## Data Availability Statement

The raw data supporting the conclusions of this article will be made available by the authors, without undue reservation, to any qualified researcher.

## Ethics Statement

The studies involving human participants were reviewed and approved by the ethics committee of the Institute of Physiology and Pathology of Hearing, Warsaw, Poland. Written informed consent for participation was not required for this study in accordance with the national legislation and the institutional requirements.

## Author Contributions

JK: conceptualization. WJ: formal analysis. JK, WJ, DBP, AG-S, and HS: investigation. DBP and AG-S: resources. JK and WJ: data curation and writing—original draft preparation. AG-S and HS: supervision. HS: funding acquisition. All authors contributed to the article and approved the submitted version.

## Conflict of Interest

The authors declare that the research was conducted in the absence of any commercial or financial relationships that could be construed as a potential conflict of interest.

## Publisher's Note

All claims expressed in this article are solely those of the authors and do not necessarily represent those of their affiliated organizations, or those of the publisher, the editors and the reviewers. Any product that may be evaluated in this article, or claim that may be made by its manufacturer, is not guaranteed or endorsed by the publisher.
